# Delineation of Genotype X Environment Interaction for Grain Yield in Spring Barley under Untreated and Fungicide-Treated Environments

**DOI:** 10.3390/plants12040715

**Published:** 2023-02-06

**Authors:** Vishnukiran Thuraga, Ulrika Dyrlund Martinsson, Ramesh R. Vetukuri, Aakash Chawade

**Affiliations:** 1 Department of Plant Breeding, Swedish University of Agricultural Sciences, 23422 Lomma, Sweden; 2 Husshållnigssällskåpet, Borgeby, 23791 Lomma, Sweden

**Keywords:** *Hordeum vulgare* L., grain yield, biplot, stability

## Abstract

Barley (*Hordeul vulgare* L.) is the fourth most important cereal crop based on production and cultivated area. Biotic stresses, especially fungal diseases in barley, are devastating, incurring high possibilities of absolute yield loss. Identifying superior and stable yielding genotypes is crucial for accompanying the increasing barley demand. However, the identification and recommendation of superior genotypes is challenging due to the interaction between genotype and environment. Hence, the present investigation was aimed at evaluating the grain yield of different sets of spring barley genotypes when undergoing one of two treatments (no treatment and fungicide treatment) laid out in an alpha lattice design in six to seven locations for five years, through additive main effects and multiplicative interaction (AMMI), GGE biplot (genotype + genotype X environment), and stability analysis. The combined analysis of variance indicated that the environment was the main factor that contributed to the variation in grain yield, followed by genotype X environment interaction (GEI) effects and genotypic effects. Ten mega environments (MEs) with five MEs from each of the treatments harboured well-adapted, stable yielding genotypes. Exploiting the stable yielding genotypes with discreet use of the representative and discriminative environments identified in the present study could aid in breeding for the improvement of grain yield in spring barley genotypes.

## 1. Introduction

Barley (*Hordeum vulgare* L.) is one of the most widely grown cereal crops based on cultivated area and production quantity. It is the fourth most popular cereal (146 × 10^6^ tonnes) after wheat (771 × 10^6^), rice (787 × 10^6^) and maize (1210 × 10^6^) [1], supplementing the world’s food and fodder requirements, alongside its utilization in the beer industry as raw material [2,3]. In the next five decades, the deployment of coarse grains as feed in developing countries is expected to increase, accounting for 56% of food grain demand [4], leading to increased production pressure on the cultivation of barley. To meet the increasing global food demand, the world barley production needs to be augmented by 54% in the next five decades [5]. World barley production has reached 158 × 10^6^ tonnes, with 1.5 × 10^6^ tonnes produced in Sweden [1]. Assuring food security through the evaluation, identification and development of high-yield varieties is one of the core objectives of the plant breeding program. Grain yield is a complex quantitative trait influenced by genetic and environmental factors [6,7]. However, problems arise in recommending a genotype with high yield due to the complex nature of grain yield and interactions between genetic, environmental, edaphic factors. Among these issues, genotype (G) X environment (E) interaction (GEI) is one of the major obstacles in exploiting and gaining full advantage of the genetic potential of genotypes, thereby slowing the progress of breeding [8]. The existence of GEI in cultivars can be confirmed based on noticeable disparity in the phenotypic performance of the genotypes in different environmental conditions, which arises due to variation in the genetic potential of genotypes and their ability to adapt for different environmental conditions [9]. Hence, the existence of GEI in crops will decrease the association between genotype and phenotype, leading to ambiguity in the selection and recommendation of genotypes to specific environments or locations [10]. The reduced selection efficiency of superior genotypes due to GEI could be conquered by evaluating genotypes in multiple locations/environments with the aim to identify stable, environment-specific genotypes [9,11] and attaining more stable and higher yields. The different statistical methodologies employed in dissecting the role of GEI to identify desirable genotypes in multiple environmental trials can be categorized into two types: univariate and multivariate methods. Out of all the available methods of depicting GEI, additive main effect multiplicative interaction (AMMI) and genotype + genotype × environment interaction effect (GGE) models are extensively used for their ability to detect GEI through genotype ranking across environments [12].

Achieving the targeted yield improvement is decelerated due to crop losses associated with various intrinsic and extrinsic factors, of which, diseases alone can cause crop losses of up to 20% of global production. Among all the diseases, fungal diseases have attained special attention due to their widespread nature and their ability to influence yields by anywhere from 1% to 100%, depending on the pathogen strain and host resistance to infection [13]. Disease management relies on the choices made regarding crop rotation, tillage, cultivars used, and the use of fungicides [14,15]. The quickest and most reliable measure in disease control for ensuring good yield is employing fungicides. However, the response to disease control practices such as the use of fungicides on cultivars with diverse genetic backgrounds can vary, due to their variation in sensitivity to environmental conditions and adaptation ability in different microclimate environments [16,17]. However, the increased application of fungicides in crop production is unsustainable due to the increased production costs and bio-augmentation through environmental contamination [18,19,20,21]. Hence, the identification and development of cultivars with high/stable yield across different environments with the marginal requirement of fungicides is desirable and will favour sustainable barley production. Understanding genotypic interaction with fungicide application will open avenues to lower crop production costs through limited fungicide application. da Silva et al. [21] studied the effect of fungicide-treated and untreated conditions on the yield of Brazilian oat cultivars (*Avena sativa* L.) and identified environment-specific genotypes with adaptability and stability. However, there are only a few reports on grain yield with/without fungicide application and the adaptability to different environments in barley [22,23,24]. Hence, the current investigation was aimed at studying the effect on grain yield in response to fungicide application and the identification of stable genotypes, better adapted to different locations in Sweden through AMMI and stability indices under untreated and fungicide-treated conditions.

## 2. Results

### 2.1. Mean Genotypic Performance

**Figure 1:**
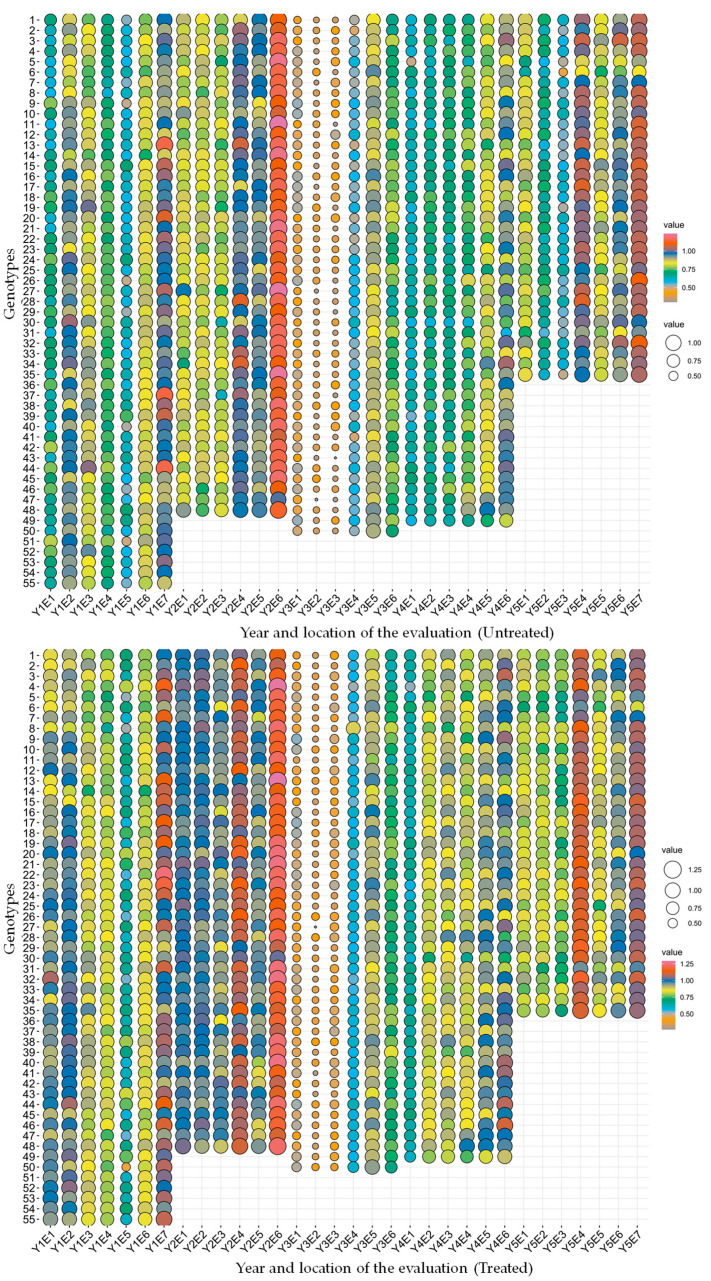
Balloon plot representing grain yield (kg m^−2^) of tested genotypes under untreated and treated conditions. The legend of colour scale value and different size balloon scale value represent the grain yield (X axis represents year and evaluated environments. Y axis represents the genotypes evaluated).

**Figure 2:**
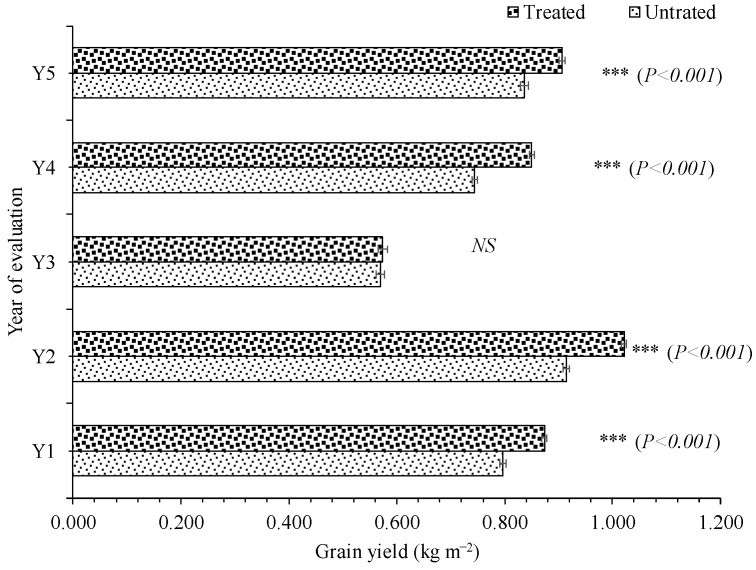
Influence of fungicide on mean grain yield (kg m^−2^) of spring barley genotypes during evaluated years. (Asterisk denotes significance at the level of *p* < 0.001 (***).

The meteorological characteristics showed wide variation in temperature, humidity and precipitation across all five years of evaluation (Table S1). The mean grain yield of genotypes varied widely, indicating substantial variation in the genotypic potential of the genotypes under evaluation (Table 1). The genotypes with the highest mean grain yields were G44 with 0.882 kg m^−2^ and 0.945 kg m^−2^ in Y1, G3 and G4 with 0.959 kg m^−2^ and 1.070 kg m^−2^ in Y2, G12 and G8 with 0.609 kg m^−2^ and 0.622 kg m^−2^ in Y3, G34 and G38 with 0.808 kg m^−2^ and 0.898 kg m^−2^ in Y4, and G32 and G3 with 0.897 kg m^−2^ and 0.950 kg m^−2^ in Y5 under untreated and treated conditions, respectively (Figure 1). The application of fungicide significantly improved the mean grain yield, and this increase was highest in Y4, followed by Y2, Y1 and Y5 (Figure 2).

### 2.2. AMMI Analysis of Variance

**Figure 3:**
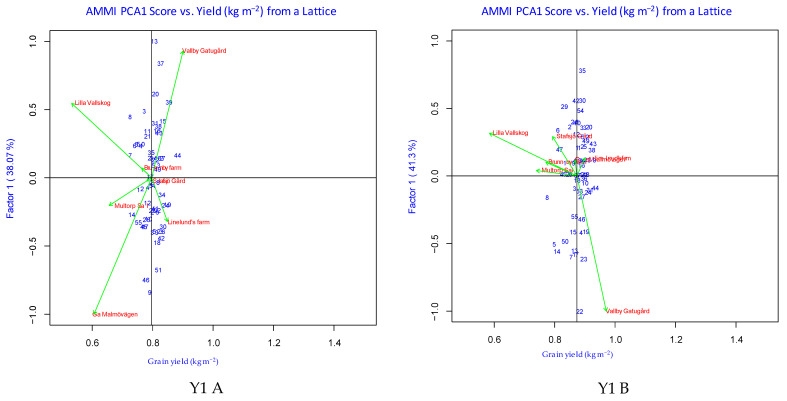

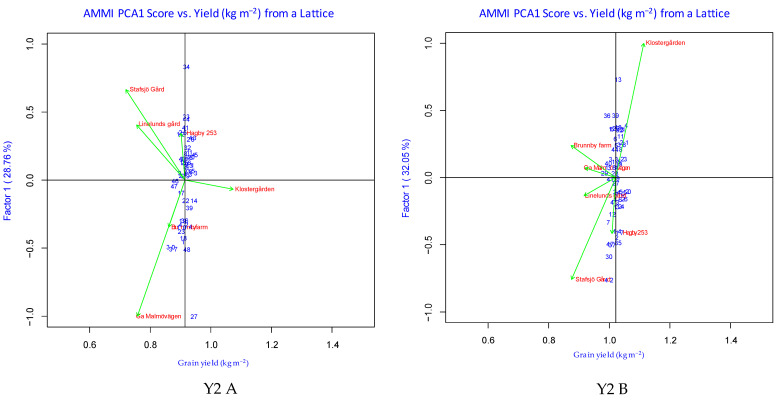

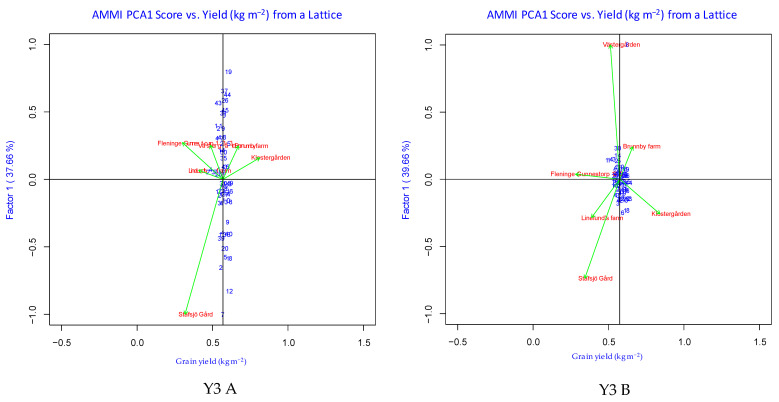

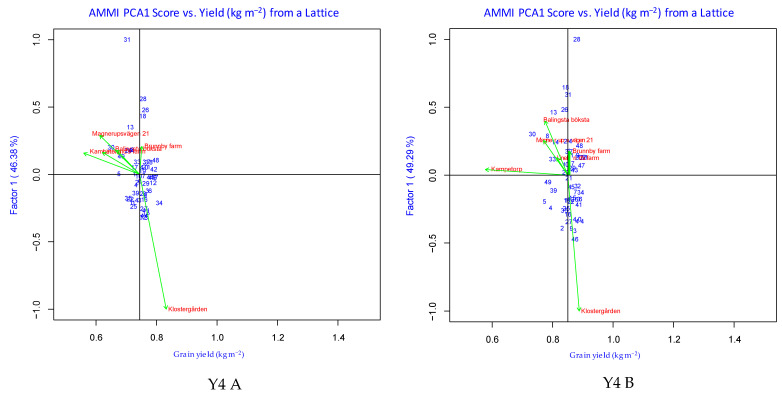

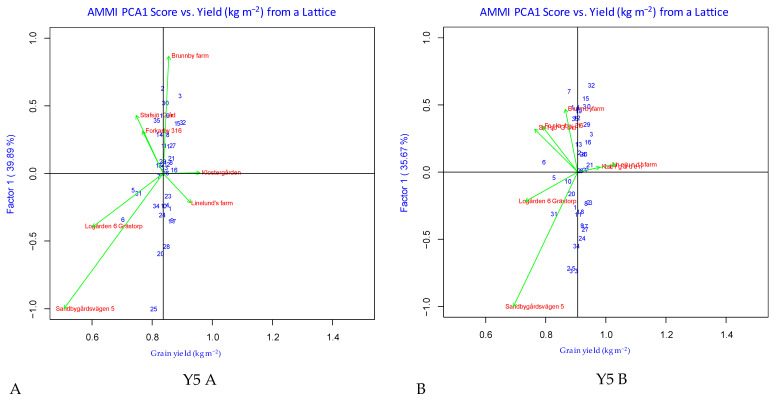
Plot of AMMI PCA1 scores of the grain yield (kg m^−2^) under untreated (**A**) and treated (**B**) conditions, across five years (Y1 to Y5).

AMMI analysis of variance for the evaluated locations in each year for five years revealed that environment is the major cause of variation in grain yield across all years, with 73.2–96.5% and 73.9–95.6% shares of the sum of squares under untreated and treated conditions. The GEI effects accounted for 2.2–17.8% and 2.7–16.5% of sum of squares under untreated and treated conditions, respectively, whereas genotypic effects captured 1.2–9.0% and 1.6–9.6% of the sum of squares under untreated and treated conditions, respectively (Table 2). The plot of the first interaction principal component axis (IPCA1) explained 28.8–46.4% and 32.0–49.3% of the sum of squares under untreated and treated conditions, respectively, while the IPCA2 revealed 19.2–27.2% and 19.9–30.1% shares of the sum of squares under untreated and treated conditions, respectively. IPCA1 + IPCA2 explained 55.9–69.3% and 55.6–73.0% of the GEI sum of squares for grain yield under untreated and treated conditions, respectively (Table 2). The plot of the IPCA1 scores for grain yield of genotypes across locations classified the genotypes into four categories based on the mean grain yield: genotypes with higher grain yield showing positive interaction effect (Quadrant I) or negative interaction effect (Quadrant IV), and the genotypes with lower grain yield showing positive interaction effect (Quadrant II) or negative interaction effect (Quadrant III) (Figure 3). The details of the genotypes classified into each quadrant are given in Table 3. Among the tested environments, E6 (both untreated and treated) in Y1, E6 (untreated) and E2 (treated) in Y2, E5 (untreated) and E2 (treated) in Y3, E1 (both untreated and treated) in Y4 and E7 (untreated) and E2 (treated) in Y5 had the lowest IPCA2 scores, with even lower IPCA1 scores. Among all the evaluated genotypes, six genotypes (G17, G23, G34, G41, G53 and G54) in Y1, ten genotypes (G3, G47, G4, G39, G36, G21, G24, G19, G35 and G14) in Y2, eight genotypes (G1, G8, G14, G27, G32, G33, G46 and G49) in Y3, ten genotypes (G1, G2, G6, G7, G10, G12, G19, G29, G37 and G46) in Y4 and 12 genotypes (G1, G4, G5, G12, G16, G23, G24, G27, G29, G31, G33 and G34) in Y5 had lower IPCA1 and IPCA2 values under untreated conditions. Conversely, under fungicide-treated environments, nine genotypes (G3, G9, G10, G16, G24, G31, G34, G37 and G48) in Y1, nine genotypes (G10, G15, G18, G25, G27, G33, G34, G41 and G45) in Y2, five genotypes (G13, G44, G26, G45 and G48) in Y3, eight genotypes (G15, G19, G23, G32, G33, G34, G43 and G49,) in Y4 and 12 genotypes (G4, G5, G8, G10, G11, G13, G16, G18, G22, G23, G28 and G29) in Y5 showed lower IPCA1 and IPCA2 values. The grain yields of the genotypes with low IPCA1 and IPCA2 scores ranged from 0.525 to 0.959 kg m^−2^ and from 0.567 to 1.032 kg m^−2^ under untreated and treated conditions, respectively (Table S2). Among the identified genotypes with low IPCA scores, between two and seven genotypes in each evaluated year had a higher grain yield (1–8%) than the mean grain yield. One genotype each in Y1 (G34) and Y4 (G19) and five genotypes (G4, G5, G16, G23 and G29) in Y5 were commonly identified as stable, with good grain yield across untreated and treated conditions (Table S2).

### 2.3. Environmental Delineation

**Figure 4:**
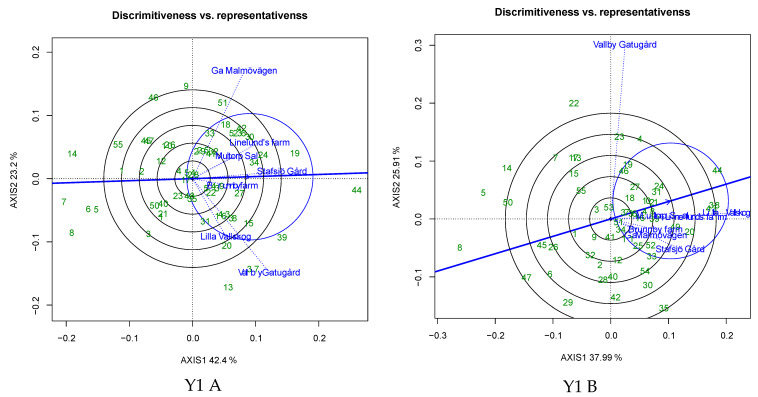

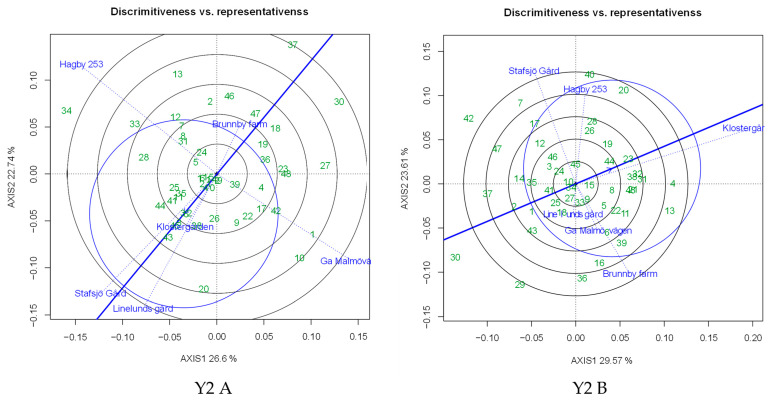

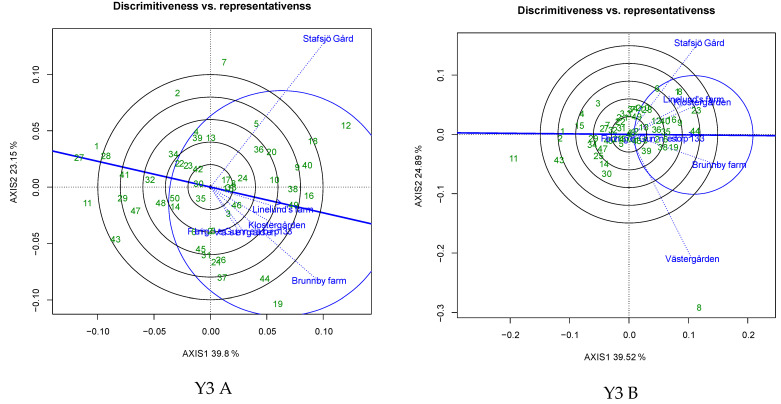

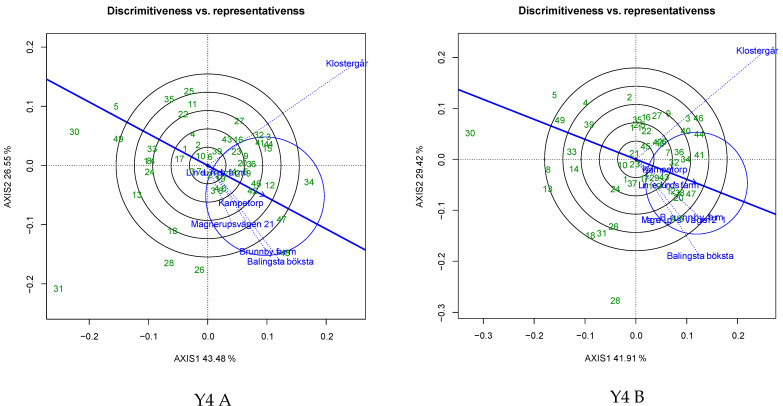

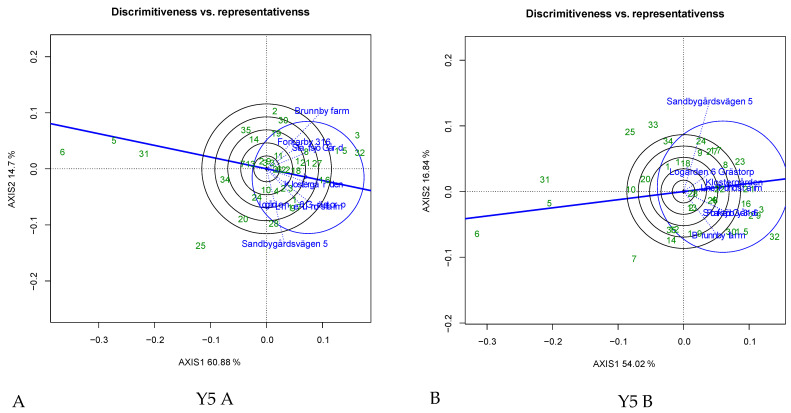
GGE biplot for barley genotypes under untreated (**A**) and treated (**B**) conditions based on environment-focused scaling for the comparison of the tested environments based on average environment axis (AEA), across five years (Y1 to Y5).

Evaluation of spring barley genotypes for grain yield revealed that the average environmental mean values under untreated conditions varied between 0.602 kg m^−2^ (E5) and 0.971 kg m^−2^ (E7) in Y1, between 0.789 kg m^−2^ (E3) and 1.144 kg m^−2^ (E6) in Y2, between 0.369 kg m^−2^ (E2) and 0.883 kg m^−2^ (E5) in Y3, between 0.629 kg m^−2^ (E1) and 0.902 kg m^−2^ (E6) in Y4 and between 0.577 kg m^−2^ (E3) and 1.029 kg m^−2^ (E7) in Y5 (Figure 3). Conversely, under treated conditions, the yield ranged from 0.654 kg m^−2^ (E5) to 1.041 kg m^−2^ (E7) in Y1, from 0.944 kg m^−2^ (E5) to 1.184 kg m^−2^ (E6) in Y2, from 0.350 kg m^−2^ (E2) to 0.911 kg m^−2^ (E5) in Y3, from 0.647 kg m^−2^ (E1) to 0.958 kg m^−2^ (E6) in Y4 and from 0.762 kg m^−2^ (E3) to 1.102 kg m^−2^ (E4) in Y5 (Figure 3). The angle between the environmental vectors was less than 90° for E2-E3-E4-E6 in Y1, E2-E3-E6 in Y2, E1-E2-E4-E5-E6 in Y3, E1-E2-E3-E4-E5 in Y4, and E1-E2-E4-E5-E7 in Y5 under untreated conditions (Figure 4A); and E1-E2-E3-E4-E5-E6 in Y1, E1-E2-E5 in Y2, E1-E2-E3-E5-E6 in Y3, E1-E2-E3-E4-E5 in Y4, and E1-E2-E4-E5-E6-E7 in Y5 under treated conditions (Figure 4B). Among all the tested environments, E7-E1-E3 in Y1, E1-E4-E2-E3 in Y2, E3-E6-E1-E5 in Y3, E6-E4-E5 in Y4 and E6-E3-E4 in Y5 were highly discriminating under untreated conditions (Figure 4A). Under fungicide-treated conditions, E7-E5-E2 in Y1, E3-E5-E6 in Y2, E4-E3-E6-E5 in Y3, E6-E4-E5 in Y4 and E3-E6-E5 in Y5 were highly discriminative (Figure 4B). The average environmental axis (AEA) of the GGE Biplot designated the most representative environment to be E3 in Y1, E3-E6 in Y2, E1-E5 in Y3, E1-E3-E4-E5 in Y4 and E7-E4 in Y5 under untreated conditions (Figure 4A). Under fungicide-treated conditions, E4-E5-E2 in Y1, E6 in Y2, E2-E6-E5 in Y3, E1-E3-E5 in Y4 and E4-E7 in Y5 were the most representative environments (Figure 4B).

### 2.4. Genotypic Potential and Stability Indices

**Figure 5:**
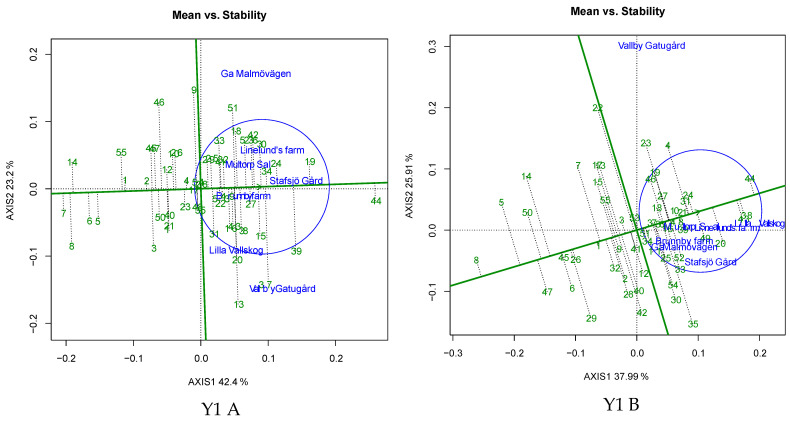

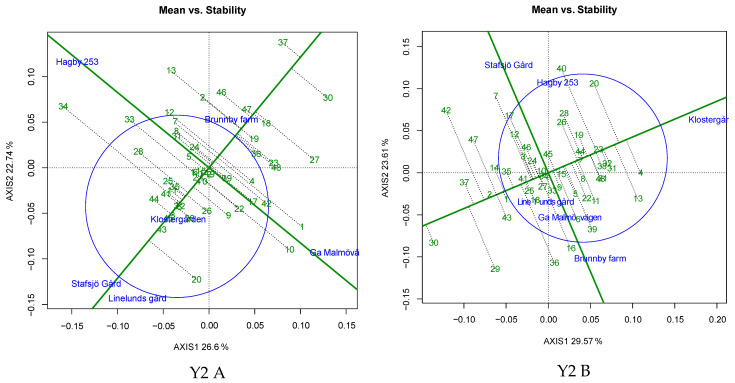

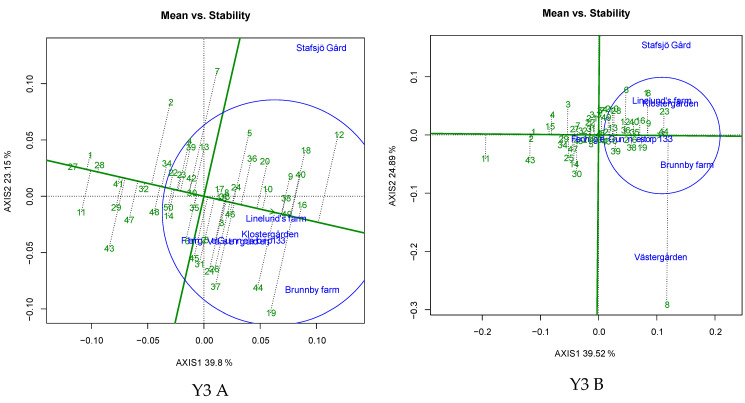

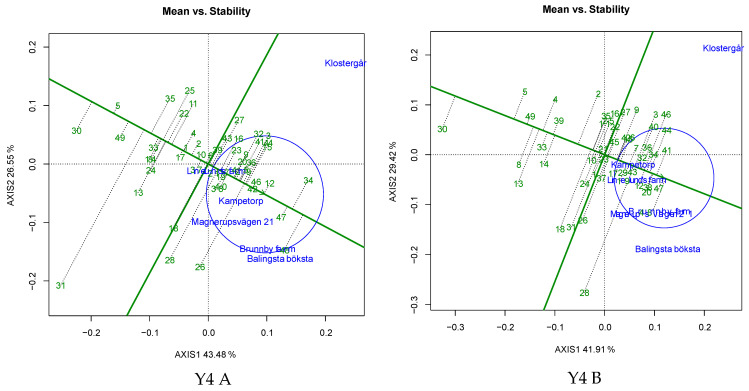

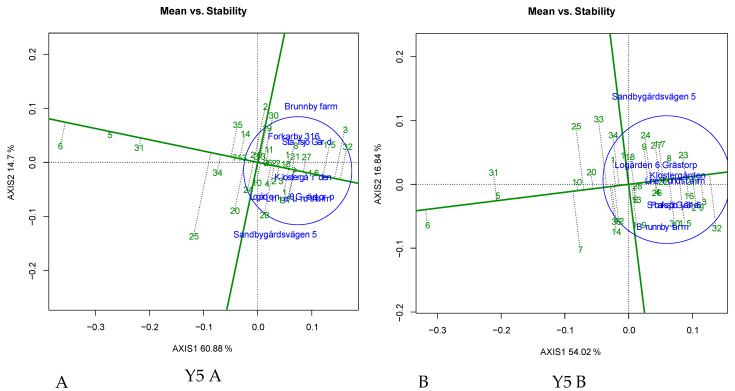
Average environment coordination (AEC) plots of barley genotypes for the mean genotypic performance and stability under untreated (**A**) and treated (**B**) conditions, across five years (Y1 to Y5).

A total of 24 genotypes in Y1, 14 genotypes in Y2, 18 genotypes in Y3, 24 genotypes in Y4 and 16 genotypes in Y5 had positive genotypic potential (GP) under both untreated and treated conditions. The genotypic potential (GP) values, AMMI stability values (ASV) and genotypic selection index (GSI) values across all evaluated years are presented in Table S2. The average environmental coordinate (AEC) axis of the biplot recommended the stable genotypes under untreated (eight in Y1, eleven in Y2, fourteen in Y3, twenty in Y4, and eight in Y5) and treated conditions (fourteen in Y1, seven in Y2, twenty-two in Y3, ten in Y4, and six in Y5) (Figure 5). Stable genotypes, according to AEC with positive GP values accompanied by superior stability, were identified under untreated and treated conditions (Table S3), with grain yield ranging from 0.571 to 0.959 kg m^−2^ and from 0.576 to1.035 kg m^−2^ under untreated and treated conditions, respectively (Table 4).

**Figure 6:**
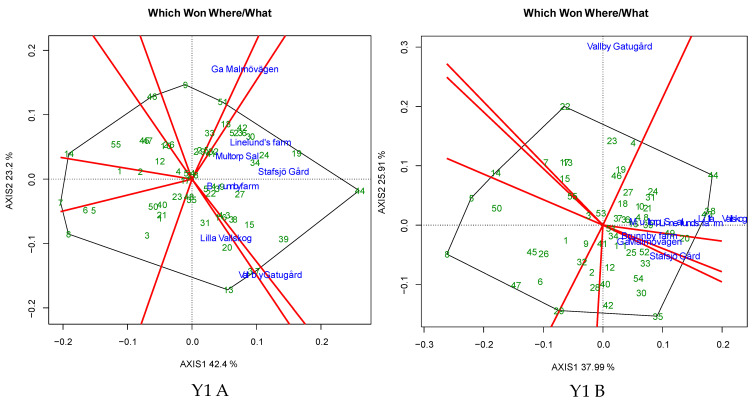

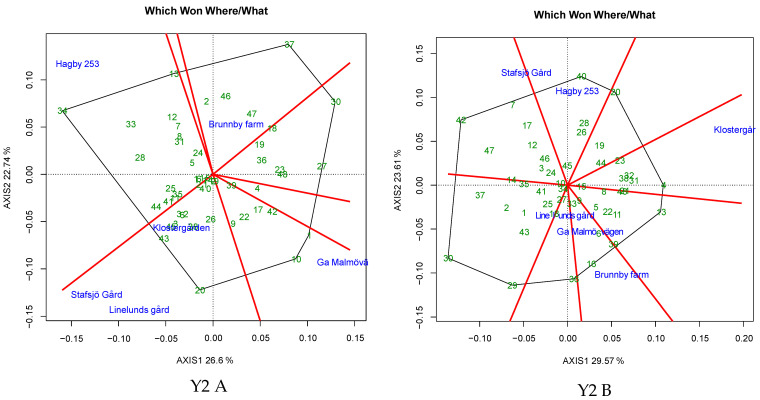

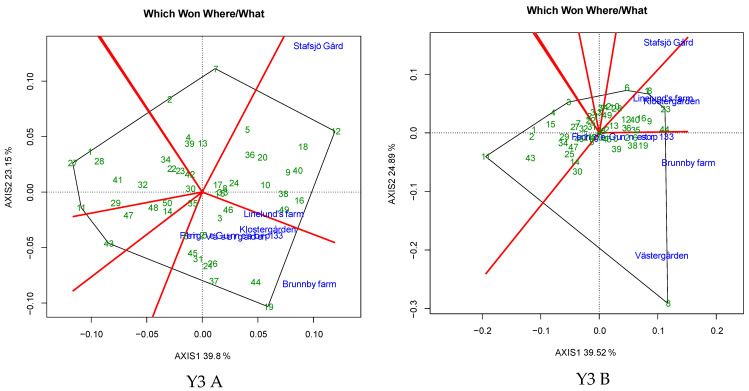

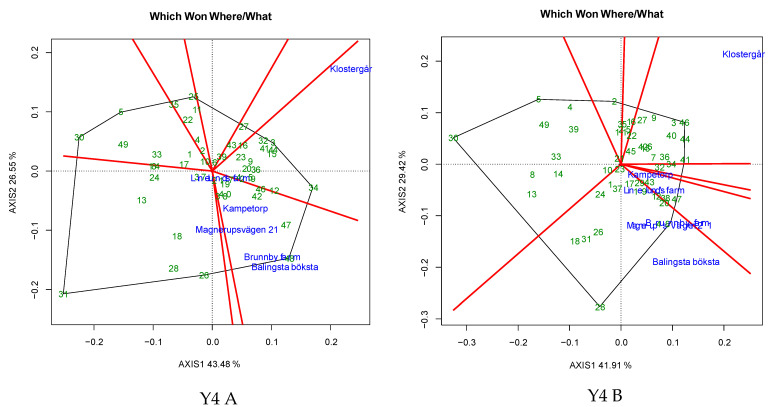

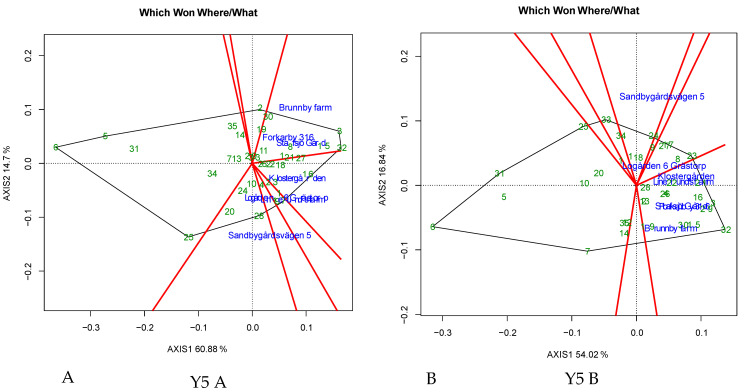
Which-won-where (WWW) plots of barley genotypes and environments under evaluation, indicating mega environments (MEs) and winning genotypes under untreated (**A**) and treated (**B**) conditions, across five years (Y1 to Y5).

The process of identifying the most suitable genotype for each environment and locating the mega environments was executed using a which-won-where plot. The polygon view of the biplot partitioned the genotypes into six to nine sectors, and the environment distribution of the sectors indicated the presence of mega-environments (MEs) (Figure 6). In Y1, the ME under untreated conditions comprised of E2, E3, L4 and E6, and under the treated condition, E2, E4 and E5 formed an ME, with G44 as the winning genotype under both untreated and treated conditions. In Y2, the ME was represented by E2, E3 and E6 under untreated conditions and by E1, E2 and E5 under treated conditions, with G20 as the winning genotype under untreated conditions. In Y3, the ME was formed by E2, E4, E5 and E6 under untreated conditions, with G19 as the winning genotype, whereas E2, E4 and E6 formed an ME under treated conditions, with G8 as the winning genotype under treated conditions. In Y4, the ME was represented by E1, E2, E4 and E5, with G48 as winning genotype, under untreated conditions, while E2, E4 and E5, with G28 as the winning genotype, formed the ME under treated conditions. In Y5, E1, E5 and E6 formed an ME under untreated conditions, with G3 as the winning genotype, whereas E1, E4, E5, E6 and E7 formed an ME under treated conditions, with G32 as vertex genotype. Each ME harboured between three and eighteen genotypes under untreated and treated conditions, with specifically adapted genotypes across both conditions with one to three common environments (Table 5). Among all the tested genotypes, seven genotypes in Y1 (G24, G27, G36, G38, G39, G43 and G44), one genotype in Y2 (G6), three genotypes in Y3 (G19, G26 and G46), one genotype in Y4 (G19) and four genotypes in Y5 (G3, G15, G30 and G32) were commonly identified to be in MEs under untreated and treated conditions (Table 5). Among the well-adapted genotypes from MEs, two genotypes in Y1 (G49, G53), three genotypes in Y2 (G3, G29, G38), one genotype in Y3 (G3), and eight genotypes in Y4 (G7, G19, G21, G38, G40, G42, G46, G47) manifested good stability under untreated condition, while seven genotypes in Y1 (G10, G16, G21, G36, G37, G44 and G48), five genotype in Y3 (G10, G17, G26, G46 and G50), one genotype in Y4 (G17) and three genotypes in Y5 (G21, G22 and G28) exhibited stability under treated conditions.

## 3. Discussion

Barley breeding with a focus on developing high-yield and admissibly stable genotypes is challenged by the varied performance of the genotypes under different locations or environments. Genotype evaluation in multiple environments and the identification of the best performing genotype lacks efficienct selection due to the interaction of genotypes with the environment, thereby reducing the correlation between the phenotype and genotype, leading to ambiguity in identifying the best performing genotype. Along with GEI, fungal diseases are one of the major problems facing barley cultivation, causing substantial yield losses, which could be managed through fungicide administration. Moreover, genotypic interaction with the fungicides, along with the confounding effect of GEI, toughens the process of discerning promising genotypes. Therefore, the present investigation was undertaken to identify high-yield and stable barley genotypes under untreated and fungicide-treated conditions using AMMI-GGE biplot analysis, which could aid in the reduced usage of fungicides, thus increasing sustainable production. Employing AMMI and GGE biplot approaches in understanding GEI is considered to be a systematic approach for grouping the genotypes in accordance with the environment through ranking based on the phenotypic performance and for understanding the relationships between the tested genotypes and environments [25,26,27,28]. The results of this experiment revealed the complex nature of grain yield and the confounding effects of fungicides, such as significant improvement of the mean grain yield in all tested years, except Y3. The preliminary economic analysis suggests that fungicide spraying resulted in a 4.5% increase in malting barley profits with substantial yield improvement [22]. However, fungicide application always does not translate into yield improvement, which could be explained by the variation in the magnitude of disease influence on some genotypes [22,29]. In the current investigation, the non-significant differences in yield recorded in Y3 might be associated with the low humidity due to diminished rainfall, making it unfavourable to disease incidence. The genotypes evaluated under the current study revealed significant differences in grain yield across all years, indicating the existence of genetic differences in yield under untreated and fungicide-treated conditions. The results of the analysis of variance from AMMI indicated that a major portion of the sum of squares of grain yield under untreated and treated conditions can be attributed to location, followed by GEI and genotype (Table 2). In the current investigation, location accounted for the largest share of sum of squares, indicating the diverse nature of environments and that a major part of grain yield variation was due to variation in location. Similar findings have been reported previously [9,30,31,32]. The application of the AMMI model for the decomposition of GEI effects revealed that the combination of IPCA1 and IPCA2 together explained 55.9–69.3% and 55.6–73.0% of total GEI under untreated and treated conditions, respectively, and the scores of IPCA1 and IPCA2 revealed 32 and 29 stable genotypes across all the years in untreated and treated conditions, respectively (Figure 6). IPCA1 and IPCA2 scores are a depiction of the genotypic stability across the environments; genotypes with low scores are expected to have high stability across all the tested environments. The use of both IPCA1 and IPCA2 is a strong approach for the identification of stable genotypes since it allows for conclusions about consistency in genotypic performance and their divergence, along with the role of the environment [33]. 

**Figure 7:**
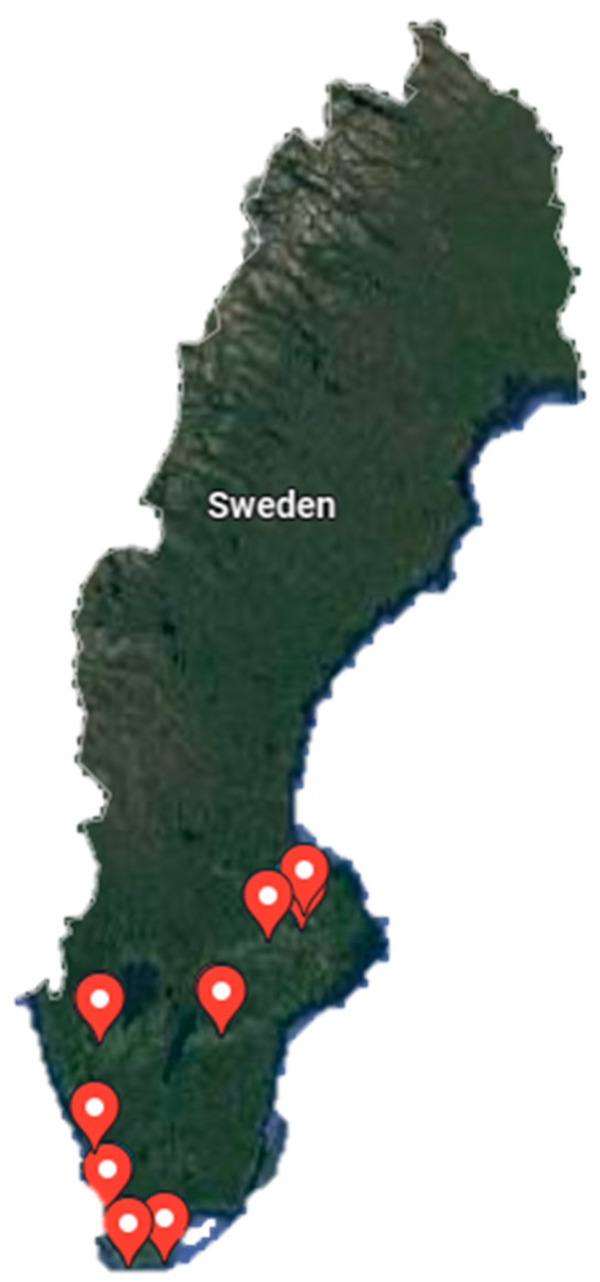
Map showing experimental trial locations in Sweden (Courtesy source from Google Maps 2022 (Google, accessed on 12 September 2022)). The red sign posts in the map indicates the geographical location of evaluated environments.

The angle between the environment vectors conveys the association among the evaluated environments [34,35,36]. In the current investigation, the angle between the environments in untreated (three to five environments/year) and treated (three to six environments/year) conditions was less than 90°, inferring a positive association among the environments. Delineation of the evaluated environments into groups based on the cosine of the environmental vector angle has been reported previously in barley [28,31,37,38]. Among all the tested environments, E3 in Y1, E2 in Y2, E1 and E5 in Y3, E4 and E5 in Y4, and E5 in Y5 were highly discriminative and representative environments under untreated conditions. Under treated conditions, E2 and E5 in Y1, E6 in Y2, E5 and E6 in Y3, and E5 in Y4 were highly discriminative and representative. The test environment efficiency is evaluated based on discrimination and representation ability [37]. The discrimination ability of an environment is revealed by the length of environmental vectors, where the length of each vector is directly proportional to the standard deviation of the environment itself [25]. In the present investigation, highly discriminative environments with good representativeness under untreated and treated conditions were the candidates for delineating the broadly adapted genotypes, while the discriminative and non-representative environments identified were better suited to studying genotypes with special adaptability [39]. Among all the tested genotypes in the present study, 50% to 61% of genotypes represented the positive genotypic potential for grain yield under untreated and treated conditions in each year, indicating their superior performance with respect to grain yield. Ndiaye et al. [40] used the genotypic potential index to identify the better performing sorghum genotypes with respect to grain yield and biomass. Among the tested genotypes, three to twelve genotypes in every evaluated year and treatment had a smaller perpendicular line to the AEC axis of the biplot (Figure 6), inferring the stability of genotypes. Similar results were reported by Kendal et al. [28] in barley. Based on the AEC, ASV and GSI indices, twelve genotypes in Y1, seven genotypes in Y2, fifteen genotypes in Y3, nineteen genotypes in Y4 and eight genotypes in Y5 were identified as demonstrating stable performance under all tested environments (Table 4). The ASV indicates the stable genotypes (with ASV values near to 0 indicating stability) based on the balanced measures from the sum of square values of IPCA1 and IPCA2, whereas GSI index integrates the ASV with the grain yield of the genotypes, thereby further increasing the selection efficiency for better genotypes. ASVs are commonly used in studies for the identification of stable barley genotypes under multiple environmental studies [7,11,41]. Among the common stable genotypes, G53 (Y1), G3 (Y2), G8 (Y3), G38 (Y4) and G33 (Y5) under untreated conditions and G16 (Y1), G44 (Y3), G43 (Y4) and G28 (Y5) under treated conditions manifested lower IPCA1 and IPCA2 values along with higher yields, indicating their stability across the evaluated environments. Similar results were reported by Elakhdar et al. [42] in barley under salt stress conditions. Which-won-where analysis of the biplot identified mega environments comprising of three to five locations in each evaluated year (Figure 7), allowing breeders to identify good test environments for the detection of genotypes adapted for the specific environmental factors [39,43,44]. In the present investigation, within each year, locations were partitioned into different MEs, and the pattern of grouping was different between untreated and treated conditions, with one to three common environments between the untreated and treated conditions, which infers that these common environments are suitable for assessing the adapted genotypes under both untreated and fungicide-treated level evaluations. WWW analysis of biplots is the most efficient way of delineating the GEI of genotypes through plotting the multi-location data of environments and genotypes in a polygon view of a GGE biplot [45]. In the present study, WWW plots revealed that G44 (Y1 untreated and treated), G20 (Y2 untreated), G19 (Y3 untreated), G8 (Y3 treated), G48 (Y4 untreated), G28 (Y4 treated), G3 (Y5 untreated) and G32 (Y5 treated) were the vertex genotypes, with higher yields in each ME. WWW plots of GGE biplots is an efficient method of determining the best genotypes in mega environments [42]. The superior-yield, winning genotypes identified in the MEs could be considered as checks in fungicide evaluation trials within the evaluated environments [46]. Because of the significant contribution of location to the variation in grain yield, the ideal genotype identified in multi-environment evaluation should have high performance, combined with stability across environments. In the present investigation, genotypes suitable for multiple environments with stable performance were identified under both untreated and treated conditions in Y1 (G49 and G53 in untreated condition, and G10, G16, G21, G36, G37, G44 and G48 in treated condition), Y2 (G3, G29 and G38 in untreated condition), Y3 (G3 in untreated condition and G10, G17, G26, G46 and G50 in treated condition), Y4 (G7, G19, G21, G38, G40, G42, G46 and G47 in untreated condition and G17 in treated condition) and Y5 (G21, G22 and G28 in treated condition). Similarly, da Silva et al. identified stable and better-adapted oat genotypes for yield and grain quality under untreated and fungicide-treated conditions [21]. Vaezi et al. evaluated barley genotypes for three years and identified stable genotypes based on stability statistics and the GGE biplot approach [9].

## 4. Materials and Methods

### 4.1. Experimental Site and Plant Material

The study was executed with different sets of spring barley genotypes in seven locations over five years (2016–2020) under the Sweden National Trails program with two different treatments (untreated and treated with fungicide). A diverse set of spring barley genotypes were evaluated each year at seven locations and a new set of genotypes were used each year, as per the updated list of released/popularly cultivated genotypes (Table S4). The five different years under genotypic evaluation were denoted as Y1 (2016), Y2 (2017), Y3 (2018), Y4 (2019) and Y5 (2020). In each year, genotypes were evaluated at six to seven different locations in Sweden (Figure 7). Location descriptions along with meteorological data of each environment are presented in Table S1, and the meteorological data were obtained from https://sverigeforsoken.se/s (Accessed on 03 September 2021). All the spring barley genotypes were raised in alpha design with two replications in each environment, using standard agronomic practices except for fungicide application. Treatment was imposed by the application of an extra dose of fungicide to determine the variety of fungal resistance. During Y1, Flexity (Flexity^®^, from BASF), Proline EC250 (Proline EC250, from BAYER) and Comet Pro (Comet^®^ Pro, from BASF) were applied for the treatment plot in all locations, and during Y2, Flexity, Siltra Xpro (Siltra Xpro from BAYER), Comet Pro was applied. In Y3, Y4 and Y5, Talius (Talius^®^ from Corteva Agriscience), Siltra Xpro and Comet pro were applied to maintain treatment. During physiological maturity, the crop was harvested for grain yield, and the data were reported as grain yield kg/square meter (kg m^−2^).

### 4.2. Statistical Analysis

The grain yield data of the spring barley genotypes were assessed for stability and G X E interaction using the AMMI model with GGE biplots under control and elevated fungicide treatment environments using GEA-R (GEA-R, CIMMYT, Mexico) [47]. The AMMI analysis has been found to be reliable in capturing a large proportion of G X E sum of squares, which clearly separates the main effects and interaction effects. Hence, it is ordinarily the first choice model when both the main effects and interaction effects are important, which is the usual case with the yield trials [48,49].

GGE is a linear-bilinear model, which is recommended when the environments are the main source of variation in relation to the contributions of genotypes and GEI with respect to the total variability. At the same time, this technique allows the determination of mega-environments (GEA-R, CIMMYT, Mexico).

The model employed for AMMI and GGE analysis is given below, and the results of the analysis were presented in the form of biplots.

AMMI analysis:

$$Y_{ij}=µ+g_{i}+e_{j}+\overset{N}{\underset{n=1}{\sum }}\tau_{n}\;\Upsilon_{in}\;\delta_{jn}+\varepsilon_{ij}$$

GGE analysis:

$$Y_{ij}=µ+e_{j}+\overset{N}{\underset{n=1}{\sum }}\tau_{n}\;\Upsilon_{in}\;\delta_{jn}+\varepsilon_{ij}$$

*Y_ij_* represents the yield of the *i*th genotype in *j*th environment; grand mean, genotype and environment deviations from grand mean are represented by µ, *g_i_* and *e_j_*. *τ_n_* represents the eigenvalue of principal component (PC) analysis axis *n*. The number of PCs and error terms are denoted by *N* and *ε_ij_*.

Analysis of variance of the grain yield data was performed using open software R [50] with the agricolae package [51]. The genotypic potential (GP) index was calculated according to Ndiaye et al. [40] by employing the formula below. A genotype with a positive GP value indicates good genotypic potential and vice versa for a negative value.

$$\mathrm{Genotypic}\;\mathrm{potential}\;\mathrm{index}=\frac{Y_{\dot{I}j}-\bar{Y}}{\bar{Y}}$$

*Y_ij_* represents grain yield of a given genotype *i* in a given environment *j*, while $\bar{Y}$ denotes overall mean grain yield.

The AMMI stability values (ASV) were calculated using the method formulated by Purchase et al. [52] via the following formula.

$$\mathrm{Ammi}\;\mathrm{stability}\;\mathrm{value}\;\left(\mathrm{ASV}\right)=\sqrt{{\left[\frac{{\mathrm{SS}}_{{\mathrm{IPCA}}_{1}}}{{\mathrm{SS}}_{{\mathrm{IPCA}}_{2}}}\left({\mathrm{IPCA}}_{1}\right)\right]}^{2}+{\left({\mathrm{IPCA}}_{2}\right)}^{2}}$$

where SS in the equation denotes the sum of squares of the first (IPCA1) and second (IPCA2) interaction principal components, and the genotypic scores are obtained from the AMMI model.

The genotype selection indexes (GSIs) of the evaluated genotypes in the present investigation were calculated using the following formulae as obtained from Farshadfar and Sutka [53].

GSI*_i_* = *Y_i_* + ASV*_i_*

where GSI*_i_* refers to genotype selection index of the *i*th genotype; *Y_i_* refers to rank of mean grain yield of *i*th genotype and ASV*i* denotes the rank of ASV of *i*th genotype. 

## 5. Conclusions

The present investigation revealed that the grain yields of barley genotypes are largely affected by location, followed by GEI and genotypes. Fungicide application significantly increased the grain yield and altered genotypic stability. Genotypes adapted to multiple environments manifesting stable yield were identified under untreated (G49 and G53 in Y1; G3, G29 and G38 in Y2; G3 in Y3; and G7, G19, G21, G38, G40, G42, G46 and G47 in Y4) and treated (G10, G16, G21, G36, G37, G44and G48 in Y1; G10, G17, G26, G46 and G50 in the treated condition in Y3; G17 in Y4; and G21, G22 and G28 in Y5) conditions, which are possible candidates for the molecular dissection and further yield improvement of spring barley in the targeted locations. The MEs and winning genotypes (G44 in Y1, G20 in Y2, G19 and G8 in Y3, G48 and G28 in Y4, and G3 and G32 in Y5) identified in the present study advocate precise testing of germplasm for grain yield under untreated and fungicide-treated trials. Prudent use of the identified genotypes from evaluation as pre-breeding material will hold potential in the development of barley genotypes with broad adaptation and stable yield.

## Figures and Tables

**Table 1:** Variation in grain yield (kg m^−2^) across five years under treated and untreated conditions.

Year	Treatment	Grain Yield (kg m^−2^)	
		Range	Mean
Y1 (2016)	Untreated	0.724–0.882	0.797 ± 0.037
	Treated	0.774–0.946	0.874 ± 0.036
Y2 (2017)	Untreated	0.863–0.959	0.915 ± 0.041
	Treated	0.980–1.070	1.021 ± 0.031
Y3 (2018)	Untreated	0.525–0.609	0.569 ± 0.058
	Treated	0.499–0.622	0.574 ± 0.06
Y4 (2019)	Untreated	0.649–0.808	0.744 ± 0.033
	Treated	0.731–0.898	0.85 ± 0.035
Y5 (2020)	Untreated	0.702–0.897	0.837 ± 0.045
	Treated	0.793–0.950	0.906 ± 0.036

**Table 2:** AMMI analysis of variance for spring barley genotypes for grain yield under untreated and treated conditions. Asterisks following F values indicate significance at the level of *p* < 0.05 (*), 0.01 (**), 0.001 (***), while NS denotes non-significance.

Source	Year	Treatment	SS	DF	MS	F	Explained (%)
Location	2016	Treated	10.45	6	1.742	648.75 ***	80.6
	2017		4.20	5	0.841	228.01 ***	80.4
	2018		22.79	5	4.557	1756.15 ***	95.6
	2019		5.88	5	1.176	526.81 ***	73.9
	2020		6.92	6	1.153	448.01 ***	85.6
	2016	Untreated	11.78	6	1.964	647.25 ***	85.0
	2017		8.38	5	1.676	303.5 ***	87.8
	2018		21.00	5	4.200	2262.1 ***	96.5
	2019		4.99	5	0.998	341.44 ***	73.2
	2020		11.63	6	1.939	571.12 ***	89.9
Location * Genotypes	2016	Treated	1.65	324	0.005	1.9 ***	12.7
	2017		0.85	235	0.004	0.98 NS	16.2
	2018		0.65	245	0.003	1.03 NS	2.7
	2019		1.32	240	0.005	2.46 ***	16.5
	2020		0.57	204	0.003	1.08 NS	7.0
	2016	Untreated	1.27	324	0.004	1.29 **	9.2
	2017		0.97	235	0.004	0.75 NS	10.1
	2018		0.49	245	0.002	1.07 NS	2.2
	2019		1.22	240	0.005	1.73 ***	17.8
	2020		0.56	204	0.003	0.81 NS	4.3
Genotypes	2016	Treated	0.86	54	0.016	5.92 ***	6.6
	2017		0.18	47	0.004	1.03 NS	3.4
	2018		0.38	49	0.008	3.03 ***	1.6
	2019		0.76	48	0.016	7.13 ***	9.6
	2020		0.60	34	0.018	6.86 ***	7.4
	2016	Untreated	0.81	54	0.015	4.93 ***	5.8
	2017		0.20	47	0.004	0.76 NS	2.1
	2018		0.26	49	0.005	2.91 ***	1.2
	2019		0.61	48	0.013	4.37 ***	9.0
	2020		0.75	34	0.022	6.53 ***	5.8
PC1	2016	Treated	0.68	59	0.012	4.65 ***	41.3
	2017		0.27	51	0.005	1.53 *	32.0
	2018		0.26	53	0.005	2.06 ***	39.7
	2019		0.65	52	0.012	6.75 ***	49.3
	2020		0.20	39	0.005	3.02 ***	35.7
	2016	Untreated	0.48	59	0.008	3.47 ***	38.1
	2017		0.27	51	0.005	1.64 **	28.8
	2018		0.18	53	0.003	2.16 ***	37.7
	2019		0.56	52	0.011	5.62 ***	46.4
	2020		0.22	39	0.006	3.4 ***	39.9
PC2	2016	Treated	0.50	57	0.009	3.51 ***	30.1
	2017		0.23	49	0.005	1.37 NS	27.6
	2018		0.15	51	0.003	1.22 NS	22.6
	2019		0.31	50	0.006	3.38 ***	23.8
	2020		0.11	37	0.003	1.78 **	19.9
	2016	Untreated	0.28	57	0.005	2.11 ***	22.3
	2017		0.26	49	0.005	1.61 *	27.2
	2018		0.13	51	0.003	1.63 **	27.2
	2019		0.28	50	0.006	2.89 ***	22.9
	2020		0.11	37	0.003	1.73 **	19.2
PC3	2016	Treated	0.16	55	0.003	1.18 NS	9.8
	2017		0.18	47	0.004	1.1 NS	21.3
	2018		0.11	49	0.002	0.91 NS	16.2
	2019		0.17	48	0.004	1.96 ***	13.2
	2020		0.10	35	0.003	1.67 *	17.7
	2016	Untreated	0.18	55	0.003	1.38 *	14.2
	2017		0.18	47	0.004	1.19 NS	19.2
	2018		0.08	49	0.002	1.04 NS	16.7
	2019		0.18	48	0.004	1.91 ***	14.5
	2020		0.09	35	0.002	1.45 NS	15.3
PC4	2016	Treated	0.13	53	0.003	1.01 NS	8.1
	2017		0.11	45	0.002	0.67 NS	12.5
	2018		0.08	47	0.002	0.73 NS	12.4
	2019		0.11	46	0.002	1.33 NS	8.6
	2020		0.08	33	0.002	1.42 NS	14.2
	2016	Untreated	0.16	53	0.003	1.29 NS	12.7
	2017		0.15	45	0.003	1.01 NS	15.6
	2018		0.06	47	0.001	0.79 NS	12.3
	2019		0.12	46	0.003	1.38 NS	10.1
	2020		0.06	33	0.002	1.17 NS	11.6
PC5	2016	Treated	0.10	51	0.002	0.75 NS	5.8
	2017		0.06	43	0.001	0.37 NS	6.5
	2018		0.06	45	0.001	0.55 NS	9.0
	2019		0.07	44	0.002	0.83 NS	5.1
	2020		0.04	31	0.001	0.7 NS	6.5
	2016	Untreated	0.10	51	0.002	0.84 NS	8.0
	2017		0.09	43	0.002	0.63 NS	9.3
	2018		0.03	45	0.001	0.42 NS	6.2
	2019		0.07	44	0.002	0.87 NS	6.1
	2020		0.05	31	0.002	0.98 NS	9.1
PC6	2016	Treated	0.08	49	0.002	0.67 NS	4.9
	2017		0.00	41	0.000	0 NS	0.0
	2018		0.00	43	0.000	0 NS	0.0
	2019		0.00	42	0.000	0 NS	0.0
	2020		0.03	29	0.001	0.68 NS	6.0
	2016	Untreated	0.06	49	0.001	0.53 NS	4.8
	2017		0.00	41	0.000	0 NS	0.0
	2018		0.00	43	0.000	0 NS	0.0
	2019		0.00	42	0.000	0 NS	0.0
	2020		0.03	29	0.001	0.55 NS	4.8

**Table 3:** Genotype classification from IPCA1 scores vs. grain yield.

Year	Treatment	Quadrant	Genotypes																		
Y1 (2016)	Untreated	I	G13	G15	G16	G20	G22	G27	G31	G37	G38	G39	G43	G44	G48	G49	G53				
		II	G3	G5	G6	G7	G8	G11	G17	G21	G23	G35	G40	G50							
		III	G1	G2	G4	G9	G10	G12	G14	G26	G45	G46	G47	G55							
		IV	G18	G19	G24	G25	G28	G29	G30	G32	G33	G34	G36	G41	G42	G51	G52	G54			
	Treated	I	G11	G16	G20	G21	G25	G30	G32	G33	G34	G35	G37	G38	G39	G43	G48	G49	G51	G52	G54
		II	G1	G2	G6	G9	G12	G26	G28	G29	G40	G41	G42	G45	G47						
		III	G3	G5	G7	G8	G13	G14	G15	G17	G18	G50	G55								
		IV	G4	G10	G19	G22	G23	G24	G27	G31	G36	G44	G46	G53							
Y2 (2017)	Untreated	I	G3	G6	G7	G8	G11	G13	G20	G24	G28	G29	G31	G32	G33	G34	G35	G38	G43	G44	G45
		II	G2	G5	G9	G12	G15	G16	G21	G25	G26	G40	G41								
		III	G1	G10	G17	G18	G19	G23	G30	G36	G37	G42	G46	G47							
		IV	G4	G14	G22	G27	G39	G48													
	Treated	I	G4	G5	G8	G11	G13	G19	G21	G23	G31	G32	G38	G48							
		II	G6	G15	G16	G18	G22	G28	G29	G33	G34	G36	G39	G40	G44						
		III	G1	G7	G12	G27	G30	G37	G41	G42	G43	G46	G47								
		IV	G2	G3	G9	G10	G14	G17	G20	G24	G25	G26	G35	G45							
Y3 (2018)	Untreated	I	G3	G6	G19	G21	G25	G26	G35	G37	G44	G45	G46								
		II	G11	G14	G27	G28	G29	G31	G32	G41	G43	G47	G48	G50							
		III	G1	G2	G4	G7	G13	G15	G17	G22	G23	G30	G34	G39	G42						
		IV	G5	G8	G9	G10	G12	G16	G18	G20	G24	G33	G36	G38	G40	G49					
	Treated	I	G8	G10	G17	G19	G26	G38	G39	G46	G50										
		II	G2	G5	G11	G14	G24	G25	G29	G30	G34	G43	G45	G47							
		III	G1	G3	G4	G7	G15	G21	G22	G27	G31	G32	G33	G48							
		IV	G6	G9	G12	G13	G16	G18	G20	G23	G28	G35	G36	G37	G40	G41	G42	G44	G49		
Y4 (2019)	Untreated	I	G18	G19	G21	G26	G28	G37	G38	G40	G42	G48									
		II	G5	G8	G13	G14	G17	G24	G30	G31	G33	G49									
		III	G1	G2	G4	G6	G10	G11	G22	G25	G35	G39	G43								
		IV	G3	G7	G9	G12	G15	G16	G20	G23	G27	G29	G32	G34	G36	G41	G44	G45	G46	G47	
	Treated	I	G1	G12	G17	G19	G20	G28	G29	G31	G37	G38	G43	G47	G48						
		II	G8	G10	G13	G14	G18	G23	G24	G26	G30	G33									
		III	G2	G4	G5	G11	G16	G25	G35	G39	G49										
		IV	G3	G6	G7	G9	G15	G21	G22	G27	G32	G34	G36	G40	G41	G42	G44	G45	G46		
Y5 (2020)	Untreated	I	G3	G8	G11	G12	G15	G16	G18	G21	G22	G26	G27	G30	G32	G33					
		II	G2	G13	G14	G19	G29	G35													
		III	G5	G6	G7	G20	G24	G25	G31	G34											
		IV	G1	G4	G9	G10	G17	G23	G28												
	Treated	I	G2	G3	G4	G13	G15	G16	G19	G21	G22	G26	G28	G29	G30	G32					
		II	G6	G7	G12	G14	G35														
		III	G1	G5	G10	G11	G20	G25	G31	G33	G34										
		IV	G8	G9	G17	G18	G23	G24	G27												

**Table 4:** Details of the better-performing genotypes with positive genotypic potential (GP), manifesting stable performance for grain yield based on average environmental coordination (AEC), genotype stability index (GSI) and AMMI stability value (ASV) under untreated and treated conditions. (+ sign denotes stable performance).

	Untreated						Treated					
	Genotype	Stable Performance as per AEC	Grain Yield (kg m^−2^)	ASV	GSI	GP	Genotype	Stable Performance as per AEC	Grain Yield (kg m^−2^)	ASV	GSI	GP
Y1 (2016)	G28	+	0.802	0.206	34	0.0066	G10	+	0.899	0.077	13	0.0293
	G49	+	0.816	0.576	40	0.0238	G16	+	0.887	0.184	33	0.0158
	G53	+	0.807	0.204	28	0.0133	G21	+	0.898	0.363	33	0.0277
	G54	+	0.797	0.096	33	0.0002	G36	+	0.895	0.206	26	0.0243
							G37	+	0.879	0.134	33	0.0058
							G44	+	0.946	0.295	18	0.0823
							G48	+	0.902	0.025	10	0.0321
							G51	+	0.883	0.281	39	0.0106
Y2 (2017)	G3	+	0.959	0.099	5	0.0484	G9	+	1.035	0.189	20	0.0128
	G13	+	0.922	0.796	57	0.0079						
	G24	+	0.920	0.115	27	0.0055						
	G29	+	0.915	0.135	34	0.0006						
	G35	+	0.947	0.186	43	0.0351						
	G38	+	0.935	0.184	45	0.0225						
Y3 (2018)	G3	+	0.571	0.635	33	0.0024	G10	+	0.587	0.167	31	0.0230
	G8	+	0.576	0.101	13	0.0122	G13	+	0.587	0.079	21	0.0226
	G16	+	0.606	0.233	25	0.0646	G17	+	0.577	0.271	58	0.0046
	G33	+	0.576	0.058	35	0.0112	G26	+	0.601	0.050	9	0.0464
	G49	+	0.598	0.046	50	0.0503	G35	+	0.601	0.138	18	0.0471
							G36	+	0.595	0.169	28	0.0361
							G42	+	0.576	0.194	45	0.0031
							G44	+	0.620	0.058	4	0.0793
							G46	+	0.584	0.284	56	0.0176
							G50	+	0.582	0.235	47	0.0143
Y4 (2019)	G7	+	0.756	0.058	8	0.0161	G17	+	0.858	0.252	33	0.0090
	G12	+	0.794	0.128	19	0.0669	G21	+	0.853	0.118	28	0.0033
	G19	+	0.754	0.083	22	0.0127	G29	+	0.864	0.321	34	0.0158
	G21	+	0.761	0.185	32	0.0221	G32	+	0.879	0.171	15	0.0340
	G29	+	0.768	0.152	38	0.0323	G43	+	0.878	0.122	15	0.0331
	G37	+	0.749	0.265	56	0.0060	G45	+	0.862	0.307	34	0.0134
	G38	+	0.754	0.212	53	0.0139	G47	+	0.896	0.246	9	0.0535
	G40	+	0.752	0.200	53	0.0111						
	G42	+	0.793	0.191	54	0.0654						
	G45	+	0.779	0.207	59	0.0470						
	G46	+	0.787	0.097	52	0.0578						
	G47	+	0.788	0.484	82	0.0596						
Y5 (2020)	G16	+	0.879	0.210	17	0.0508	G21	+	0.946	0.376	9	0.0432
	G18	+	0.854	0.296	23	0.0208	G22	+	0.927	0.182	15	0.0228
	G22	+	0.846	0.678	43	0.0110	G28	+	0.915	0.169	19	0.0090
	G26	+	0.845	0.341	34	0.0098						
	G33	+	0.840	0.234	35	0.0035						

**Table 5:** Barley genotypes adapted to identified MEs in the five evaluated years under untreated and treated conditions.

Year	Treatment	Location	Genotypes																	
Y1	Untreated	E2, E3, E4, E6	G15	G19	G22	G24	G27	G30	G32	G34	G36	G38	G39	G41	G42	G43	G44	G49	G52	G53
	Treated	E2, E4, E5	G10	G16	G18	G18	G21	G24	G27	G31	G36	G37	G38	G39	G43	G44	G48			
Y2	Untreated	E2, E3, E6	G3	G6	G14	G20	G21	G26	G29	G32	G38	G40	G43	G45						
	Treated	E1, E2, E5	G6	G16	G33															
Y3	Untreated	E2, E4, E5, E6	G3	G19	G21	G25	G26	G31	G37	G44	G45	G46								
	Treated	E2, E4, E6	G10	G17	G19	G26	G30	G38	G39	G46	G50									
Y4	Untreated	E1, E2, E4, E5	G7	G19	G21	G38	G40	G42	G46	G47	G48									
	Treated	E2, E4, E5	G1	G17	G18	G19	G23	G24	G26	G28	G31	G37								
Y5	Untreated	E1, E5, E6	G3	G8	G11	G12	G15	G30	G32											
	Treated	E1, E4, E5, E6, E7	G2	G3	G4	G13	G15	G16	G19	G21	G22	G26	G28	G29	G30	G32				

## Data Availability

Not applicable.

## Supplementary Materials

The following supporting information can be downloaded at: https://www.mdpi.com/article/10.3390/plants12040715/s1, Table S1: Details of meteorological characteristics of the environments under evaluation; Table S2: Details of IPCA scores of the genotypes evaluated across the environments and treatments; Table S3: Details of stability indices of the evalauted genotypes; Table S4; List of the genotype names evaluated in the present investivation.
